# Study of the Awareness of Adoption as a Family-Building Option Among Oncofertility Stakeholders in Japan

**DOI:** 10.1200/JGO.18.00043

**Published:** 2018-08-07

**Authors:** Eriko Shiraishi, Kouhei Sugimoto, Jason Solomon Shapiro, Yuki Ito, Keiko Kamoshita, Atsuko Kusuhara, Takayuki Haino, Tomoe Koizumi, Aikou Okamoto, Nao Suzuki

**Affiliations:** ^1^ The Jikei University School of Medicine, Tokyo, Japan; ^2^ Dokkyo University Saitama Medical Center, Saitama, Japan; ^3^ National Research Institute for Child Health and Development, Tokyo, Japan; ^4^ St Marianna University School of Medicine, Kanagawa, Japan

## Abstract

**Purpose:**

The oncofertility decision tree was developed by the oncofertility consortium as a tool to support healthcare professionals and patients through the complicated process of deciding the most appropriate fertility preservation strategy for patients with cancer. Various strategies include oocyte retrieval, oocyte donation, use of a gestational carrier and adoption. However, differences in the cultural and legal landscape present serious barriers to utilizing some of these strategies in Japan.

**Patients and Methods:**

We surveyed Japanese oncofertility stakeholders including 60 cancer survivors, 27 oncology facilities, 78 reproductive medicine facilities and 15 adoption agencies by a questionnaire to characterize awareness among oncofertility stakeholders in Japan about parenting options including adoption to inform work to establish guidelines for decision-making by cancer survivors in an oncofertility.

**Results:**

Our results indicate that oncologists and reproductive endocrinologists in Japan have an insufficient understanding of adoption that prevents them from adequately informing their patients. Japanese cancer survivors self-describe a lack in confidence in finding a suitable partner and raising a child. Contrastingly, of the 9 adoption agencies which responded, no agency included being a cancer survivor as a criterion for disqualification and 4 of 9 (44%) adoption agencies reported at least 1 adoption to a cancer survivor in the last year.

**Conclusion:**

Our work demonstrates that a cancer survivor’s medical history itself is not a hurdle to adoption and investment in patient-provider education could be a viable strategy to improve the utilization of adoption as a fertility preservation strategy in Japan.

## INTRODUCTION

Recent advances in oncology and reproductive medicine have prompted caregivers to rethink their views of fertility preservation for young survivors of cancer.^1,2^ The tipping point came in 2004, when Belgian clinician Donnez and colleagues reported a live birth after the transplantation of cryopreserved ovarian tissue.^3^ In 2006, the American clinician Woodruff coined the term oncofertility to refer to a new discipline that paired oncology with reproductive medicine. Woodruff proceeded to found the Oncofertility Consortium, building a network for oncofertility medicine throughout the United States and the world and informing medical practitioners and patients.^4^ A similar network that spanned several European countries was created. Oncofertility medicine is steadily spreading throughout the world; however, many medical practitioners in the field still face difficulties satisfactorily treating their patients. Cancer patients must make treatment decisions while mentally confronting the threats to both life and fertility; properly caring for these patients under these circumstances has proven to be challenging.Oncofertility care is further complicated by the multiple disciplines that are involved. The Oncofertility Consortium developed decision trees to reduce the complexities of decision making.^5^ Although these decision trees have reduced the confusion experienced by patients and doctors when deciding on treatment approaches, they include options with donated eggs and sperm, which are heavily restricted in the clinic in Japan.^6^ Although adoption provides a path to parenthood for patients with cancer who cannot conceive a child, or who opt out of fertility preservation, adopting a child is difficult in Japan and is uncommon.^7^ Moreover, little work has been done in Japan to characterize the awareness of adoption among oncofertility stakeholders—survivors of cancer, adoption agencies, oncologists, and reproductive endocrinologists (REIs). The level of awareness, however, must be measured to inform efforts to help oncofertility evolve properly in Japan and give survivors of cancer more parenting options.

The aim of the current study was to determine the level of awareness among oncofertility stakeholders in Japan about parenting options, including adoption, to inform future guidelines for decision making by survivors of cancer in an oncofertility context.

## PATIENTS AND METHODS

A questionnaire about adoption awareness was administered to survivors of cancer, adoption agencies, oncologists and REIs engaged in oncofertility care. The questionnaire was sent to clinicians at 27 oncology facilities and 78 reproductive medicine facilities that were registered with the Japan Society for Fertility Preservation. The questionnaire focused on knowledge of adoption and the provision of information on adoption to patients. With permission, a differently worded questionnaire was distributed to and collected from survivors of cancer at The Cancer Forum 2016 in Tokyo, Japan. Of the 55 responses received, 16 were from males and 39 were from females, whose ages ranged from 22 to 47 years of age (ie, of reproductive age). On average, males were 30.6 years old and females 35.3 years old. There were 30 patients with breast or gynecologic cancer and two patients with testicular cancer, which indicates that nine of the remaining 23 patients with cancer were female and 14 male. Participants were asked about their cancer type, current cancer status, desire to have children, whether they knew about adoption, and whether they were considering adoption. Yet another differently worded questionnaire was mailed to and returned by 15 adoption agencies that were registered as type 2 social welfare services, were available for questioning, and agreed to complete the questionnaire. Agencies were asked how many adoptions they handle per year, how many of these adoptions go to a survivor of cancer, and whether they disqualify survivors of cancer as adoptive parents. The ethics committee of our university approved this study.

## RESULTS

### Oncologists

We received completed questionnaires from 13 facilities (44.4%). In response to the question, “Do you know about adoption?”, 16.7% reported that they “Know much,” 33.3% reported that they “Know some,” and 50% reported that they “Know nothing.” Many of the doctors had a low level of awareness of adoption (Table1). In response to the question, “Do you provide information on adoption to your patients?”, 25% answered “Sometimes” and 75% answered “Never,” which suggests that most doctors do not provide information on adoption (Table 1). Many cited being “Not very aware about adoption” as the reason for not providing information, which indicates that many oncologists lack the exposure and training needed to inform patients about adoption. (Table1).

**TABLE 1:**
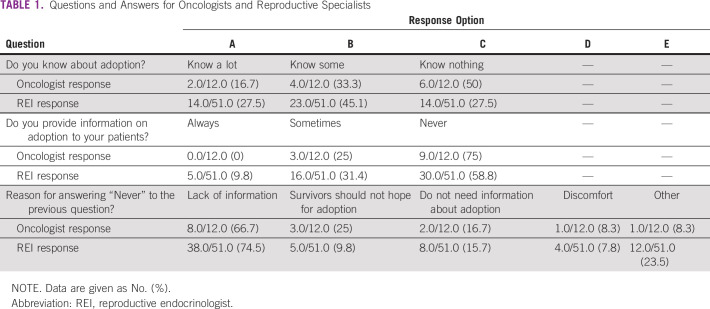
Questions and Answers for Oncologists and Reproductive Specialists

### REIs

We received completed questionnaires from 51 facilities (65.4%). Among REIs, 27.5% reported that they “Know much” and 45.1% reported that they “Know some” about adoption. Combined, these two responses amount to 72.6% of the total, which indicates that REIs have a greater awareness of adoption than oncologists (Table1). Of note, 41.2% of those REIs who responded reported providing information about adoption (Table1); thus, a majority of REIs reported not providing information, and a majority of this population cited “Not knowing very much” as the reason (Table1).

### Survivors of Cancer

**FIG 1:**
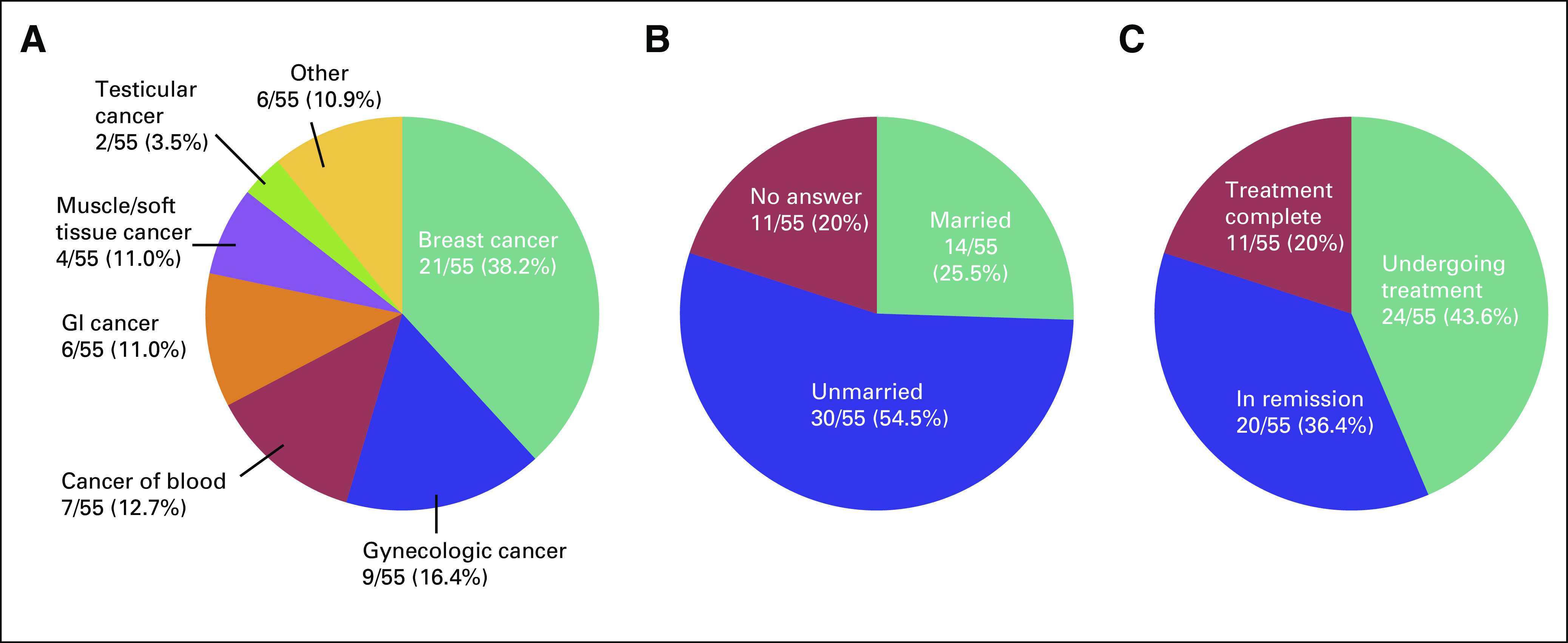
(A) Type of underlying disease of survivors of cancer. (B) Marital status of survivors of cancer. (C) Disease status of survivors of cancer.

**FIG 2:**
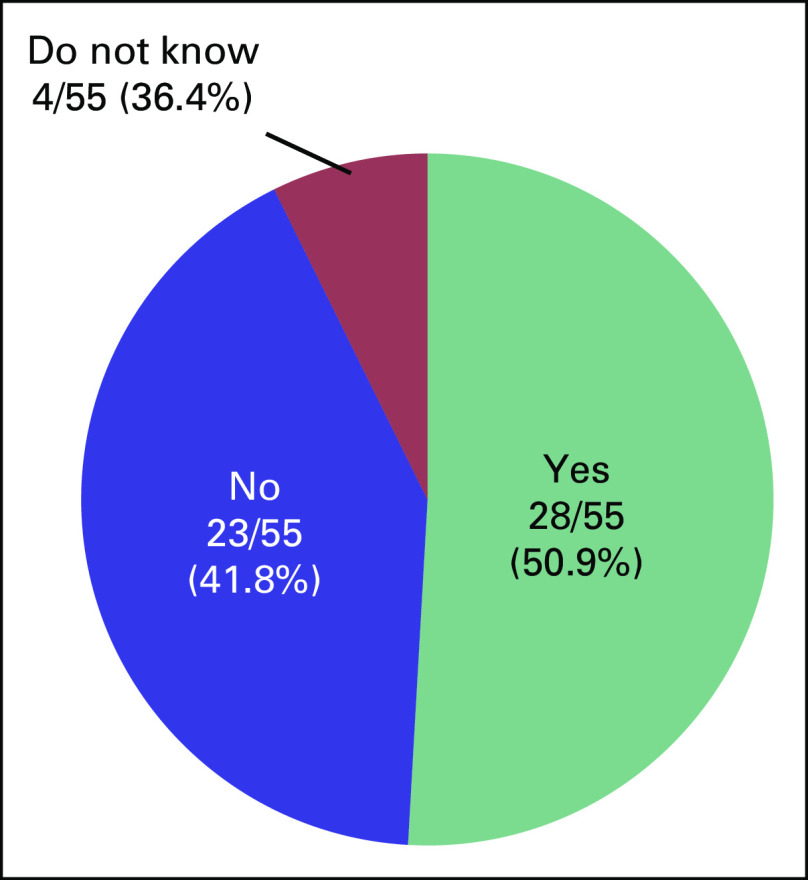
Cancer survivors’ responses to the question, “Do you want to have a child?”

**FIG 3:**
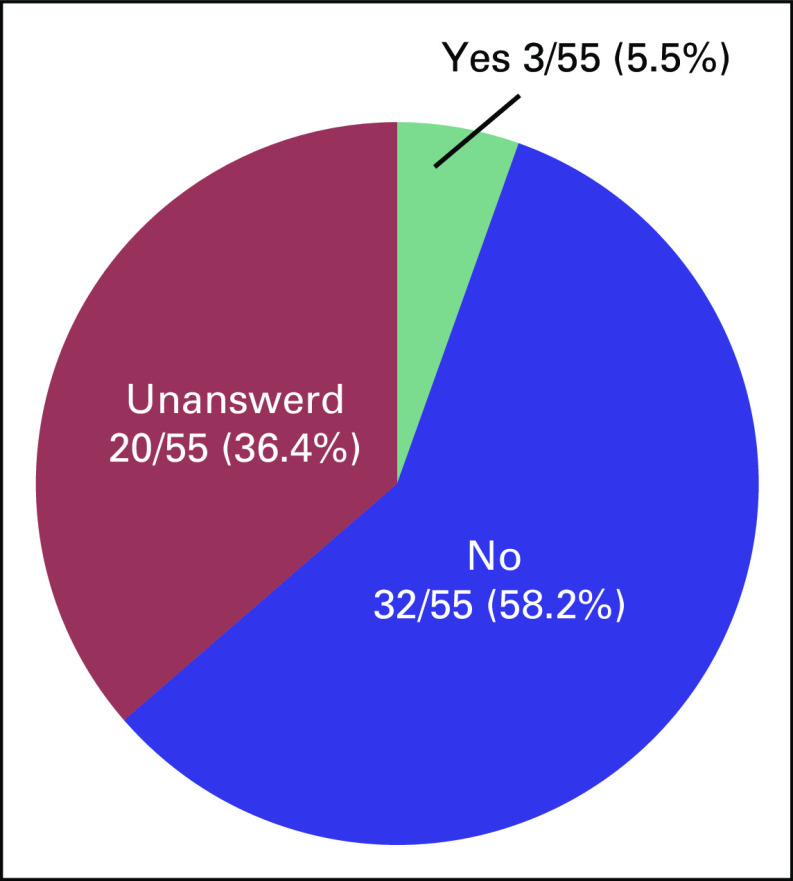
Cancer survivors’ responses to the question, “Are you considering adoption?”

We received a completed questionnaire from 55 survivors of cancer, which yielded a response rate of 91.7%. Mean age was 33.9 years. Underlying diseases, marital status, and disease status of the respondents are shown in Figures 1 to 3. Five patients had received fertility preservation before cancer therapy. Of these cases, oocyte cryopreservation was performed in two cases, embryo cryopreservation in two cases, and ovarian tissue cryopreservation in one case. No male patients received sperm cryopreservation in this survey; we have included this information. In response to the question, “Do you want to have a child?”, 50.9% of respondents answered “Yes” and 41.8% answered “No” (Fig 2). The most common reason cited for answering “No” was “Because I am unmarried,” and other common reasons were “Am unsure about being able to be in a relationship, much less married” and “Am unsure about my ability to be a parent because I am a cancer survivor” (Table 2). Unfortunately, we did not obtain information regarding the presence or the number of children from each responder; however, only four of 23 patients who specifically did not wish to have children cited already having children as the reason. Therefore, we can infer that having children before the present disease state was not a major barrier. Respondents who reported wanting to have a child were asked, “Are you considering adoption?” Only 5.5% of patients responded “Yes,” with the majority (58.2%) answering in the negative (Fig 3). Common reasons given for answering “No” included “I wish to have a biologically related child,” “Am unsure about my ability to be a parent since I’m a cancer survivor,” and “Am unsure about my ability to be a parent” (Table 3). These responses indicate that survivors of cancer tend to be anxious about their medical history and apathetic about parenting.

**TABLE 2:**
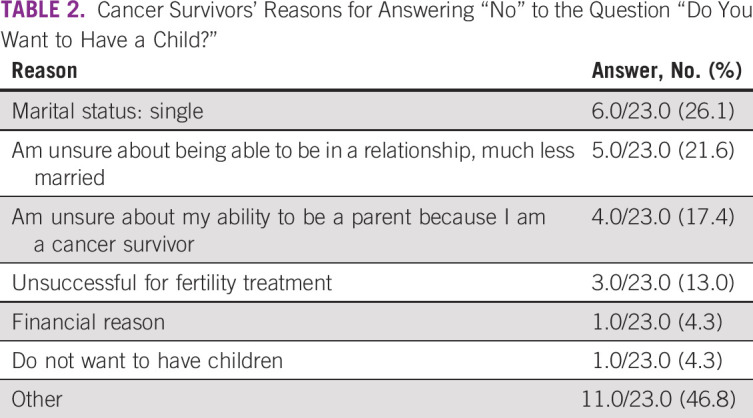
Cancer Survivors’ Reasons for Answering “No” to the Question “Do You Want to Have a Child?”

**TABLE 3:**
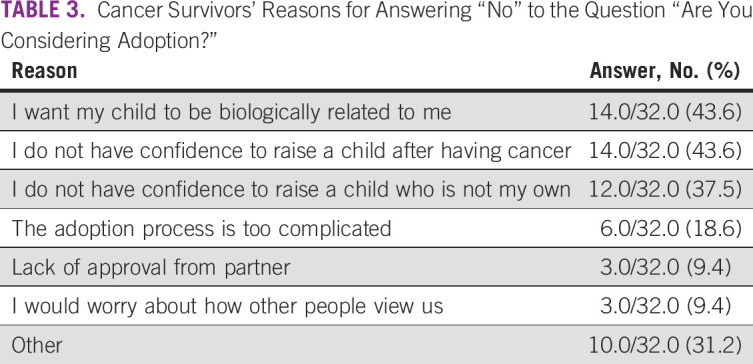
Cancer Survivors’ Reasons for Answering “No” to the Question “Are You Considering Adoption?”

### Adoption Agencies

We received a completed questionnaire from nine of 15 agencies (60%). The nine adoption agencies handled an average of 13.56 adoptions per year; however, five of the agencies brokered no adoptions for a survivor of cancer per year, two agencies brokered one adoption, one agency brokered two adoptions, and one agency brokered 10 adoptions (Table 4). Thus, few agencies brokered many successful adoptions for survivors of cancer and the numbers varied from agency to agency. All responding agencies answered “No” to the question, “Do your adoptive parent disqualification criteria include being a cancer survivor?” This demonstrates that being a survivor of cancer in Japan does not disqualify one from being an adoptive parent.

**TABLE 4:**
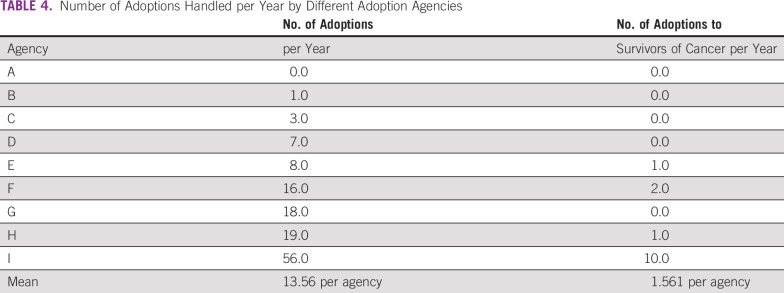
Number of Adoptions Handled per Year by Different Adoption Agencies

## DISCUSSION

Our questionnaires were designed to ascertain awareness among oncofertility stakeholders in Japan about parenting options, including adoption, to inform work to establish guidelines for decision making by survivors of cancer in an oncofertility context. In 2014, there were 512 adoption cases classified as special adoptions under Japanese law (when a child is removed from the family register of the biologic parents and becomes legally exclusively the child of the adoptive parents).^8,9^ This greatly underperforms the adoption rate in most other countries^7^ and has not been considered in any scientific publications to our knowledge.

The current study demonstrates that oncologists and REIs in Japan have an insufficient understanding of adoption, which prevents them from adequately informing their patients. It also indicates that survivors of cancer lack the self-confidence to even contemplate wanting children because of their medical history and are in an emotional state that prevents them from actively pursuing a relationship or marriage. In the United States, where adoptions are numerous, survivors of cancer were seldom able to become adoptive parents until recently.^10^ Now cancer-friendly adoption services exist, meaning that the agencies have expressed a willingness to work with prospective parents who have a history of cancer history.^11^ Our study shows that adoption agencies are willing to consider survivors of cancer as candidates for adoption on terms that are equal to those used for the general public provided the survivors meet parenting criteria; this finding may encourage survivors of cancer who lack the confidence to pursue adoption. Another factor working against adoption by survivors of cancer is the bloodline mentality of Japan, which is indicated by the answer “I want a biologically related child” that some gave for not wanting to adopt. Ito et al^12^ found the probability of a survivor of cancer being able to produce offspring after cryopreservation or a similar procedure to be 0.66. Stakeholders in Japan must consider adoption as another key option for survivors of cancer who want a child; however, investigators must first seek ways to reduce prejudice against adoption in Japanese society and make adoption more accepted. Adoption was not always commonplace in the United States. Previously, foster care was the norm, and children under foster care could always be returned to the biologic parents. However, intensifying neglect and abuse of children who were returned to their biologic parents prompted the enactment of the Adoption and Safe Families Act^13^ in 1997 under then-President Bill Clinton. This shifted adoption policy toward a stance of checking early on whether children were able to live well with their biologic parents. The act paid incentives to states that shifted from foster care to adoption, reduced the tax burden on adoptive families, and provided other benefits. This prompted many states to increase the number of foster children who were eligible for adoption.^14^ The United States also has many guidelines that instruct educators about what problems foster and adopted children face in the classroom as well as how to deal with those problems on the basis of the concept that foster and adoptive families are one of a wide variety of family situations.^15^

One of the questions survivors of cancer were asked was worded as follows: “Do you want to become an adoptive parent?” Perhaps more respondents would have answered affirmatively had the question been worded “Would you want to become an adoptive parent if you learned you were unable to have a biologically related child?” Gorman et al ^16^ reported that interest in adoption was twice as high among young female survivors of cancer than the general population. Approximately 8% of the general population in the United States receives some form of training about adoption, but only approximately 1% adopt.^17,18^ Whereas the current study was limited to survivors of cancer, similar questionnaires should be administered to the general population or infertile people in Japan. Differences in education status can have a profound effect on an individual’s or a family’s decision-making process. Although we did not directly collect information on educational background, Japan is a rather unique country in that disparities in educational achievement are smaller than in other developed nations. As of 2010, more than 80% of the Japanese population completed secondary education and literacy rates in Japan are 99%.^19^ Therefore, it is likely that Japanese oncofertility stakeholders are able to fully use relevant information on adoption or other fertility services if presented with these materials. Careful assessment of educational achievement should be included in follow-up studies.

The current study demonstrates the need for health care professionals, and reproductive specialists in particular, to learn more about adoption and the importance of informing survivors of cancer who wish to adopt that their medical history is not a hurdle. However, adoption must be reconsidered through education about family diversity, media campaigns to spread this information, and better government policy, in addition to greater awareness of these findings. This would increase awareness of adoption, which could give survivors of cancer more options in seeking relationships and marriage and ultimately improve their quality of life among.
